# Self-compassion, body appreciation, and eating disorder symptoms among young adults with weight loss goals: a diary study

**DOI:** 10.1007/s40519-025-01735-2

**Published:** 2025-03-22

**Authors:** Rachel Batchelor, Bree O’Leary, Sarah J. Egan, Cecilie Thøgersen-Ntoumani

**Affiliations:** 1 University of Oxford, Oxford, UK; 2 enAble Institute and School of Population Health. Curtin University, Perth, Australia; 3 University of Southern Denmark, Odense, Denmark; 4 Danish Centre for Motivation and Behaviour Science, Institut for Idræt Og Biomekanik, Department of Sports Science and Clinical Biomechanics, Syddansk Universitet, Odense, Denmark; 5 School of Sport, Exercise and Rehabilitation Sciences, University of Birmingham, Birmingham, UK

**Keywords:** Self-compassion, Body appreciation, Eating disorders, Eating psychopathology, Young adults, Diary study

## Abstract

**Purpose:**

Research has shown that self-compassion and body appreciation are associated with less disordered eating. However, previous literature has mostly examined between-person associations, which do not account for fluctuations in these constructs within individuals over time. Understanding such fluctuations could inform future tailored interventions to better address individuals’ needs by targeting times when they are most vulnerable. The aim of this study was to examine dynamic within-person associations between self-compassion, body appreciation, and disordered eating in young adults who self-reported trying to lose weight.

**Methods:**

Fifty-seven 18–30-year-old adults aiming to lose weight reported their levels of self-compassion, using items from the Short Form Self-Compassion Scale (SCS-SF), body appreciation, using items from the Body Appreciation Scale-2 (BAS-2) and disordered eating, using items derived from previous diary research, twice daily over a 1-week period. Generalised Linear Mixed Modelling (GLMM) was used with three levels: observations (level 1), nested within days (level 2), nested within individuals (level 3). Each GLMM included three nominal random effects (participant, day, time of day) and controlled for age, gender, and Body Mass Index (BMI). Effect sizes, represented by squared semi-partial correlation coefficients (sr2), were calculated for each GLMM via multiple linear regression analyses to represent the unique amount of variance explained by each predictor variable.

**Results:**

The findings showed that after controlling for age, BMI, and gender, negative associations between self-compassion and disordered eating, and body appreciation and disordered eating were found, while a positive association was found between self-compassion and body appreciation.

**Conclusions:**

The findings suggest that further research should examine whether fostering self-compassion and body appreciation may be putative targets to reduce disordered eating in young adults who aim to lose weight.

**Level of evidence:**

Level III: Evidence obtained from well-designed cohort or case control analytic studies.

## Background

The prevalence of dieting among young adults is high, with a large longitudinal study finding that by mid-young adulthood 59.0% of females and 34.5% of males had reported dieting in the past year [1]. Body image concerns are also common among young adults, with body dissatisfaction increasing during the transition to young adulthood [2]. Dieting, including restrictive eating, and body image concerns have been associated with nutritional deficiencies, unhealthy relationships with food, increased depression and poorer quality of life [3–5]. Exercising as a compensatory strategy to control weight and shape has also been associated with adverse mental health outcomes [6]. While particularly prevalent among individuals with a high Body Mass Index (BMI) [2, 6, 7], substantial proportion of individuals in a healthy BMI range also experience such difficulties [6, 8, 9]. As a result, it is paramount to explore factors that can protect young adults from disordered eating, operationalised in the current study as thoughts of, and engagement in, dietary restraint and exercise to influence weight or body shape [10], practices.

In recent systematic reviews, self-compassion has been proposed as a promising approach to mitigate body image concerns and disordered eating [11, 12]. Self-compassion involves taking an understanding and accepting stance towards oneself [13]. According to Neff [13], self-compassion is comprised of three key components: (a) self-kindness, which involves treating oneself with patience and care during personal failures instead of self-criticism; (b) mindfulness, which entails observing difficult thoughts and emotions without becoming overly absorbed in them (overidentifying); and (c) common humanity, which means recognising that suffering is a shared human experience rather than an isolating one. Self-compassion has been associated with fewer eating disorder behaviours [11, 12]. Self-compassion can facilitate adaptive weight management [14], and intuitive eating (i.e., eating in response to biological cues of hunger), which tends to be a more sustainable strategy than dietary restriction for managing weight [15]. It has also been postulated that self-compassion may facilitate emotion regulation, thereby reducing the use of dysfunctional attempts to regulate emotions in eating disorders (e.g., binge eating or restricting) [12]. In addition, self-compassion is associated with body appreciation [16], defined as accepting and respecting one’s body.

Body appreciation is an important indicator of positive body image [17]. While body image research has typically focused on identifying strategies that may mitigate negative body image, emphasis on promoting adaptive body attitudes (e.g., body appreciation) may have more practical utility [17]. While efforts designed to reduce negative body image may help individuals tolerate their bodies, promoting positive body image involves actively cultivating an appreciative and respectful view of one’s body [17]. Moreover, body appreciation appears to be a protective factor against eating pathology. For example, a longitudinal study with adult women found that self-compassion may reduce eating pathology and binge eating by increasing unconditional permission to eat (e.g., eating foods that are desired in a given moment, refusal to dichotomise foods as ‘good’ or ‘bad’), which is an indicator of intuitive eating [18].

An important limitation of the research investigating the associations between self-compassion, body appreciation, and disordered eating is that most studies have employed cross-sectional designs using between-subject analyses. Therefore, fluctuations in these constructs within individuals over time have not been accounted for. Understanding such fluctuations could inform future tailored interventions to better address individuals’ needs by targeting times when they are most vulnerable. Diary methodologies are helpful as they enable observations to be yielded in natural settings and as they occur, enhancing ecological validity and reducing memory recall biases [19]. By employing this methodology, it is possible to distinguish between trait-like between-person differences and dynamic within-person associations. Some research has shown that both self-compassion [14, 20, 21] and body appreciation [21] fluctuate on a daily basis, including among adults who are overweight or obese [22]. For example, in a once daily diary study, individuals with bulimia nervosa have been observed to have lower binge eating and compensatory behaviours when self-compassion is higher [20]. However, we are aware of only one diary study examining the daily associations between self-compassion, body appreciation and disordered eating. Kelly and Stephen [21] examined self-compassion, body appreciation and dietary restraint in college women for 7 days, finding that on days when participants reported more self-compassion, they also reported higher body appreciation and lower dietary restraint. However, participants only completed diary surveys once daily limiting the number of data points and thus, missing fluctuations in the variables that occur within the same day. Employing diary studies that examine the constructs more than once per day addresses this limitation and can reduce recall bias.

A further gap in the literature is that most diary studies examining different combinations of self-compassion, body appreciation and disordered eating have not controlled for BMI. This is important given that research has shown consistent positive associations between BMI, body dissatisfaction, and disordered eating [21, 23]. Although Kelly and Stephen [21] did control their analyses for BMI, this variable was self-reported, and thus prone to social desirability effects.

The primary aim of the current study was to examine within-person associations between self-compassion, body appreciation, and disordered eating among young adults with weight loss goals. Controlling for age, gender, and objective BMI, it was hypothesised that, at times when participants reported higher self-compassion, they would report less disordered eating and higher body appreciation. In addition, we hypothesised that when participants reported higher body appreciation, they would also report lower disordered eating.

## Methods

### Research design

The study comprised three phases. Phase 1 utilised a correlational survey design, Phase 2 comprised a laboratory-based assessment, and Phase 3 employed a twice daily diary study.

### Participants

Participants were recruited from [BLINDED FOR REVIEW] University undergraduate psychology research participation scheme and social media through study advertisements. The inclusion criteria were: (i) 18–30 years and (ii) have weight loss goals, defined as self-reporting trying to lose weight, and (iii) own a smartphone. The exclusion criteria were: (i) a self-reported current or prior eating disorder diagnosis, (ii) clinically relevant Eating Disorder Examination Questionnaire (EDE-Q) [24] scores (defined as 2.8 or over) [25] and/or (3) BMI < 18.5 (i.e., ‘underweight’).

According to Maas and Hox [26] a minimum of 30 participants at the between-person level are needed to achieve satisfactory statistical power for multilevel modelling (MLM) [26]. To account for attrition rates and missing observations, 58 participants were recruited, with one not completing the diary phase due to obtaining a BMI < 18.5.

The final sample comprised 57 participants (*M*_age_ = 25.28, SD = 3.09; 70.18% female, 29.82% male). Participants were mostly female and Caucasian (Table 1). Secondary school (i.e., year 12) was the highest level of education completed for over half of participants. The mean BMI of the final sample was 24.81 (SD = 3.53, range = 19.19–36.78), with most being classified into the 18.5 to 24.9 range. The mean EDE-Q overall global score was 1.42 (SD = 0.77, range = 0.16–2.54).

**Table 1:** Sociodemographic information and body mass index (BMI) of participants (*N* = 57)

Demographic	*n* (%)
Gender	
Female	40 (70.18%)
Male	17 (29.82%)
Ethnicity	
Caucasian	38 (66.67%)
Asian Pacific Islander	15 (26.32%)
European	2 (3.51%)
Aboriginal/Torres Strait Islander	1 (1.75%)
Other	1 (1.75%)
Highest level of education	
Secondary school	30 (52.63%)
University bachelor’s degree	16 (28.07%)
Postgraduate degree	10 (17.54%)
No response	1 (1.75%)
BMI	
18.5 to 24.9	33 (57.89%)
25 to 29.9	21 (36.84%)
30 to 39.9	3 (5.26%)

### Procedure

Ethical approval was granted by the [BLINDED FOR REVIEW] University research ethics committee (HRE2019-0345). Prospective participants were directed to a hyperlink to read the participant information sheet and provide electronic informed consent. Participants (*N* = 58) were then directed to the Phase 1 questionnaire, hosted on Qualtrics, which contained demographic questions and the Eating Disorder Examination-Questionnaire (EDE-Q) [24].

In Phase 2, participants (*N* = 58) attended a university laboratory to measure height and weight. A singular height measurement was obtained with their shoes removed using a stadiometer, and three consecutive weight measurements were taken by the researcher using an electronic scale. During this appointment, participants were briefed on the diary study (Phase 3) and given instructions for timely completion.

During Phase 3, participants (*N* = 57) received a hyperlink to the diary survey, via text message, twice per day, morning and evening, every day for a 7-day period (14 measurement points in total). The iPhone application ‘Scheduled’ facilitated automatic text message alerts. Participants received the link to the morning diary at random between 10 and 11am and the evening diary between 6 and 7 pm. Participants were instructed to complete each survey within an hour of receiving the alert. Responses yielded beyond this time frame were excluded. Each diary survey contained measures of state self-compassion, state body appreciation, and disordered eating. Items from these measures were randomised to ensure ordering and practice effects did not bias responses.

### Measures

#### Body mass index (BMI)

Participants had height in metres (using a SECA stadiometer) and weight in kilograms (measured three times using a FitBit Aria weighing scale) measured the day prior to commencing their diary component. Objective BMI was calculated by aggregating a mean weight from the three measurements taken and dividing the mean weight (kgs) by each participant’s respective height (m), squared.

#### Eating disorder examination-questionnaire (EDE-Q)

As part of the screening process for study eligibility, eating disorder symptoms were assessed using the EDE-Q [24]. The EDE-Q comprises 28 items assessing symptoms over the past 28 days. Responses were made using a 7-point Likert scale (0=no days to 6=every day). A mean overall global score (0–6) was calculated, with higher scores indicating greater disordered eating symptomology. A clinical cut-off score of 2.8, which has been supported as optimal for screening for eating disorders [25], was used. The EDE-Q demonstrated excellent internal consistency in the present sample (*α* = 0.93).

### Diary measures

When employing diary methodologies, particularly those comprising multiple assessments per day, it is customary to reduce the number of items per scale, if items are derived from a larger scale, to reduce participant fatigue [27, 28]. Subsequently, each diary measure was reduced to four items, informed by prior research [29–31]. Furthermore, the stem for the items contained within the measures below was adapted to refer to the time period since the last assessment (e.g., “since I woke up this morning” for morning assessments; “since my last diary entry” for evening assessments). Participants responded to the survey questions concurrently in the order self-compassion, then body appreciation, and then disordered eating. All questions for the diary measures are displayed in Supplementary Information.

*Self-compassion.* To measure self-compassion, four items were selected from the 12-item self-report Short Form Self-Compassion Scale (SCS-SF) [30]. Item selection was guided by prior factorial validation analyses [30], including the highest-loading items that were of most relevance on a daily level. Participants responded to each item by indicating how they had treated themselves in difficult times (e.g., “When I have failed at something important to me, I became consumed by feelings of inadequacy”, “I tried to keep my emotions in balance”). Responses were made using 5-point Likert scales (1 = almost never to 5 = almost always; reverse scored). An overall mean score (1–5) was then calculated, with higher scores indicating greater self-compassion. The SCS-SF has good criterion-related validity [30]. Items used in the present study had acceptable internal consistency of *α* = 0.75.

*Body appreciation.* Body appreciation was assessed using four items from the 10-item unidimensional Body Appreciation Scale-2 (BAS-2) [31]. Item selection was guided by prior factorial validation analyses [29, 31], including the highest-loading items that were of most relevance on a daily level. Participants self-reported the frequency they identified with the items (e.g., “I felt good about my body”) using 5-point Likert scales (1 = never to 5 = always). An overall mean score (1–5) was then calculated, with higher scores indicating greater body appreciation. Items used in the present study had excellent reliability (Cronbach *α* = 0.95).

*Disordered eating*. Four items derived from previous diary research [29] assessing dietary restraint and exercise to influence weight and body shape were embedded within the diary to measure engagement in disordered eating on a daily level. Using 5-point Likert scales (0 = *Not at all* to 5 = *Very much*), two items assessed thoughts about restricting eating (“Have you thought about restricting the amount of food you eat to influence your weight or shape?”) and engage in exercise to influence or control weight or shape (“Have you thought about exercising as a means of controlling your shape or amount of fat, or burning off calories?”). Two items (using the same rating scale as above) assessed actual dietary restraint (“Have you actually restricted the amount of food you eat to influence your shape or weight?”) and exercise (“Have you actually engaged in exercise as a means of controlling your shape or amount of fat, or burning off calories?”). For an overall disordered eating outcome, a mean score (0–5) was calculated, with higher scores indicating greater disordered eating. Items used in the present study had good internal reliability (*α* = 0.86).

### Data analyses

Data were analysed with SPSS version 25. Generalised Linear Mixed Modelling (GLMM) was used with three levels: observations (level 1), nested within days (level 2), nested within individuals (level 3). Each of the three GLMMs included three nominal random effects (participant, day, time of day) and controlled for age, gender, and BMI. Finally, a squared semi-partial correlation coefficient (*sr*^*2*^) indicating effect size was calculated for each GLMM via multiple linear regression analyses to represent the unique amount of variance explained by each predictor variable. GLMM is less sensitive to missing observations than traditional statistical procedures [32]. The GLMM maximum likelihood procedure is a full information estimation procedure which uses all data present within each assessment point, thus reducing the need to replace missing values [32, 33].

## Results

### Data screening and missing observations

GLMM case processing summaries indicated that the final data set comprised 658 diary entries, representing 93.6% of the maximum number of entries completed.

At least two of the three extraneous variables were found to be covariates per outcome. To ensure consistency across regression models, the three variables were treated as covariates in all three models. Table 2 shows bivariate correlations between fixed effect (i.e., extraneous) variables and exponential moving average (EMA) variables, respectively. Associations between EMA variables evidenced expected direction-specific moderate correlations [34].

**Table 2:** Mean (*M*), standard deviation (SD), and Pearson’s r correlation coefficients of fixed effect variables and EMA variables

Variable	*M*	*SD*	1	2	3	4	5	6
1. Age	25.32	3.01	–					
2. Gender	1.68	0.47	− 0.27^a^	–				
3. BMI	24.81	3.53	0.15^a^	− 0.29^a^	–			
4. Self-Compassion	3.66	0.85	0.06	0.10^a^	− 0.28^a^	–		
5. Body Appreciation	3.09	1.06	− 0.03	0.10^a^	− 0.45^a^	0.50^a^	–	
6. Disordered Eating	1.58	1.32	− 0.21^a^	− 0.01	0.23^a^	− 0.30^a^	− 0.36^a^	–

Gender: 1 = Male, 2 = Female. ^a^indicates *p* < 0.01.

Table 3 presents GLMM results of changes in self-compassion predicting changes in disordered eating. Within the model, age was a significant negative predictor of disordered eating, indicating younger participants were more likely to report disordered eating. In addition, BMI was a significant predictor of disordered eating. The relationship was positive, indicating higher BMI was associated with greater disordered eating. There was no significant effect for gender. After controlling for age, gender, and BMI, self-compassion was a significant negative predictor of disordered eating. Thus, changes in self-compassion were negatively associated with changes in disordered eating. Calculation of sr^2^ indicated that 5% of the variability in disordered eating was uniquely explained by within-person changes in self-compassion, indicating a small effect size [34].

**Table 3:** Generalised linear mixed model (GLMM) results of changes in self-compassion predicting changes in disordered eating behaviours

	CE	*SE*	*t*	Sig	95% CI	
					Lower	Upper
Intercept	3.529	0.049	71.696	**0.000**	3.432	3.625
Age	− 0.095	0.004	− 21.559	**0.000**	− 0.104	− 0.086
BMI	0.071	0.001	63.618	**0.000**	0.069	0.073
Gender = 1	− 0.118	0.079	− 1.493	0.136	− 0.273	0.037
Gender = 2	0^a^					
Self-compassion	− 0.354	0.020	− 18.074	**0.000**	− 0.393	− 0.316

CE = Coefficient estimate, SE = Standard Error, CI = Confidence Interval. Gender: 1 = Male, 2 = Female

*p* < 0.001 are in boldface. Probability distribution: Normal. Link function: Identity.

^a^This coefficient is set to zero, because it is redundant.

Table 4 presents GLMM results of changes in self-compassion predicting changes in body appreciation. Within the model, BMI was negatively associated with body appreciation. Gender was a significant positive predictor of body appreciation, indicating females reported greater body appreciation than males. There was no significant effect for age. After controlling for age, gender, and BMI, self-compassion was positively associated with body appreciation. Calculation of sr^2^ indicated 16% of the variance in body appreciation was uniquely explained by within-person changes in self-compassion, indicating a medium effect size [34].

**Table 4:** Generalised linear mixed model (GLMM) results of changes in self-compassion predicting changes in body appreciation

	CE	*SE*	*t*	Sig	95% CI	
					Lower	Upper
Intercept	3.967	0.123	32.284	**0.000**	3.726	4.209
Age	− 0.013	0.008	− 1.657	0.098	− 0.029	0.002
BMI	− 0.100	0.007	− 14.871	**0.000**	− 0.113	− 0.087
Gender = 1	0.202	0.018	11.258	**0.000**	0.167	0.238
Gender = 2	0^a^					
Self-compassion	0.520	0.021	24.859	**0.000**	0.479	0.561

CE = Coefficient estimate, SE = Standard Error, CI = Confidence Interval. Gender: 1 = Male, 2 = Female

*p* < 0.001 are in boldface. Probability distribution: Normal. Link function: Identity

^a^This coefficient is set to zero, because it is redundant.

Table 5 presents results of changes in body appreciation predicting changes in disordered eating. Age was negatively associated with disordered eating. BMI was positively associated with greater disordered eating. There were no significant effects for gender. After controlling for age, gender, and BMI, body appreciation was a significant predictor of disordered eating. The relationship was negative, indicating that changes in body appreciation were negatively associated with changes in disordered eating. Calculation of sr^2^ indicated that 7% of the variability in disordered eating behaviours was uniquely explained by within-person changes in body appreciation, indicating a small effect size [34].

**Table 5.** Generalised Linear Mixed Model (GLMM) Results of Changes in Body Appreciation Predicting Changes in Disordered Eating Behaviours

	CE	*SE*	*t*	Sig.	95% CI	
					Lower	Upper
Intercept	4.050	0.093	43.333	0.000	3.867	4.234
Age	− 0.105	0.001	− 151.766	0.000	− 0.106	− 0.103
BMI	0.053	0.001	72.357	0.000	0.052	0.055
Gender = 1	− 0.011	0.077	− 0.147	0.883	− 0.163	0.141
Gender = 2	0^a^					
Body appreciation	− 0.369	0.010	− 35.895	0.000	− 0.389	− 0.349

CE = Coefficient estimate, SE = Standard Error, CI = Confidence Interval. Gender: 1 = Male, 2 = Female

*p *< 0.001 are in boldface. Probability distribution: Normal. Link function: Identity

^a^This coefficient is set to zero because it is redundant

## Discussion

The present study examined dynamic within-person associations while controlling for between-person differences, in state self-compassion, state body appreciation, and disordered eating among young adults with weight loss goals. The results supported our hypotheses showing that participants reported less disordered eating at times when they reported higher self-compassion, consistent with the literature on trait self-compassion [11, 12]. Supporting previous findings [21], at times when participants reported more self-compassion, they also reported higher body appreciation. Furthermore, as hypothesised, when participants reported higher body appreciation, they also reported lower disordered eating.

Our findings contribute to the literature by providing preliminary support for higher self-compassion and body appreciation being associated with lower disordered eating. Such findings are important, given the potential negative effects of disordered eating previously found, such as nutritional deficiency, an unhealthy relationship with food, poor health-related quality of life and adverse mental health outcomes [3, 4, 6].

The results highlight the importance of future research exploring whether self-compassion and body appreciation play a causal and protective role against disordered eating, as this study could not establish causal effects. Indeed, Turk and Waller [12] discussed that self-compassion may reduce the use of affect regulation attempts, such as binge eating. For example, self-compassion may enable individuals to regulate their emotions [35], rather than engaging in disordered eating behaviours. However, as Turk and Waller [12] point out, our understanding of whether there is a causal role of self-compassion in eating disorders requires research, which also applies to body appreciation. Therefore, to build upon the current understanding of the protective roles of self-compassion and body appreciation, longitudinal experimental research investigating potential explanatory mechanisms (e.g., testing longitudinal mediational pathways) for their associations with disordered eating are needed.

### Clinical implications

The current study has important practical implications for informing future intervention research. Notwithstanding the non-experimental design of this study, the findings suggest that self-compassion and body appreciation may be putative targets for interventions designed to reduce disordered eating among young adults who aim to lose weight. A recent systematic review [11] reported some support for the efficacy of self-compassion interventions, particularly group interventions based on compassion-focused therapy, in reducing eating disorder symptoms among clinical eating disorder samples. In addition, self-compassion-based interventions (e.g., self-compassionate writing, self-compassionate meditation) have also been found to improve body appreciation and reduce eating disorder symptoms in clinical eating disorder and non-clinical samples [36, 37]. However, such studies have been limited by small samples, consisting of predominantly females, warranting further intervention research in larger, more diverse samples.

The current findings could also inform tailored interventions by identifying vulnerable periods when individuals are most at risk of disordered eating behaviours. By integrating EMA data into technologies like mobile apps, interventions could leverage these insights to provide timely, personalised support, such as digital prompts or techniques, during critical moments. Future research should explore the efficacy of such targeted approaches to better address individual needs.

Overall, research is required to determine whether interventions aimed at both increasing self-compassion and body appreciation are useful adjuncts to evidence-based treatments for eating disorders, and as preventive approaches.

### Strengths and limitations

There are several limitations of the current study. First, the inclusion criteria (“individuals with weight loss goals”) may have attracted body-conscious participants. Second, the sample predominantly comprised undergraduate psychology students (i.e., narrow participant pool) and individuals within a healthy BMI. Third, we did not have any individuals in the sample who identified as non-binary or gender diverse. The study, therefore, requires replication with more diverse and clinical eating disorder samples to support generalisability of the findings. Fourth, the effect sizes are small, making it important to interpret the findings in the context of these small effects. Fifth, the item-selection method employed precluded inclusion of one of the key components of self-compassion (self-kindness). In addition, the associations between each component of self-compassion and other outcomes were not considered. Future replications should include items pertaining to each component of self-compassion. Sixth, although the analyses controlled for age, gender, and BMI, other factors that previous research has found to be associated with self-compassion, body appreciation and disordered eating, such as anxiety and depression [16, 38, 39], were not considered. These constructs should be considered in future research. Finally, given the exploratory aims of the present study, alternative explanations for the findings should be considered. The observational nature of the research design precludes inferences regarding causality.

This study also has notable strengths. We extended previous research that only included female participants [e.g., [21, 40]] by including males in our sample, which is important given eating disorders occur across all genders [41]. The current study also controlled for BMI, age and gender; unlike in Kelly and Stephen’s diary study [21], BMI was measured in a controlled setting rather than self-reported in the current study, thereby reducing potential social desirability effects. The EMA enhanced ecological validity of the findings, as participants completed diary measures while in their natural environment. The randomisation of items and scales in diary measures minimised ordering effects, reducing biases inherent in repeated-measure assessments. Utilising EMA also minimised memory recall biases, with the current study addressing limitations in Kelly and Stephen’s diary study [21], by having participants complete diary measures twice daily [21]. However, future replications should consider further extending assessments per day to further reduce potential retrospective biases as well as pursue opportunities to examine fluctuations occurring within smaller time intervals than those of this study.

## Conclusion

The present study is one of only few studies to employ diary methodology to examine dynamic within-person associations between self-compassion, body appreciation, and disordered eating, and the first which has used a mixed-gender sample. The findings support the association between higher self-compassion and body appreciation and lower disordered eating. Further research evaluating the efficacy of interventions targeting self-compassion and body appreciation is warranted.

### What is already known on this subject?

Body image concerns and disordered eating behaviours are common among young adults. Self-compassion has been associated with less disordered eating and greater body appreciation, yet much of this research has examined between-person associations.

### What this study adds?

By examining dynamic within-person associations while controlling for between-person differences, this study contributes to the literature by providing preliminary support for the protective role of self-compassion and body appreciation against disordered eating in female and male young adults who aim to lose weight. Further longitudinal research investigating potential explanatory mechanisms and intervention research targeting self-compassion and body appreciation are needed.

## Data Availability

The data is available upon reasonable request and subject to institutional approvals.
